# The protein cargo of extracellular vesicles correlates with the epigenetic aging clock of exercise sensitive DNAmFitAge

**DOI:** 10.1007/s10522-024-10177-9

**Published:** 2025-01-08

**Authors:** Bernadett György, Réka Szatmári, Tamás Ditrói, Ferenc Torma, Krisztina Pálóczi, Mirjam Balbisi, Tamás Visnovitz, Erika Koltai, Péter Nagy, Edit I. Buzás, Steve Horvath, Zsolt Radák

**Affiliations:** 1 Research Centre for Molecular Exercise Science, Hungarian University of Sport Science, Alkotás U. 42-48, Budapest, 1123 Hungary; 2 Department of Molecular Immunology and Toxicology and the National Tumor Biology Laboratory, National Institute of Oncology, Ráth György U. 7-9, Budapest, 1122 Hungary; 3 Laki Kálmán Doctoral School, University of Debrecen, Nagyerdei Krt. 98, Debrecen, 4032 Hungary; 4 Chemistry Coordinating Institute, University of Debrecen, Egyetem Tér 1, Debrecen, 4032 Hungary; 5 Department of Genetics, Cell and Immunobiology, Semmelweis University, Üllői Út 26, Budapest, 1085 Hungary; 6 Institute of Organic Chemistry, HUN-REN Research Centre for Natural Sciences, Magyar Tudósok Körútja 2, Budapest, 1117 Hungary; 7 Department of Plant Physiology and Molecular Plant Biology, ELTE Eötvös Loránd University, Pázmány Péter Sétány 1/C, Budapest, 1117 Hungary; 8 Department of Anatomy and Histology, HUN-REN–UVMB Laboratory of Redox Biology Research Group, University of Veterinary Medicine, István Utca 2, Budapest, 1078 Hungary; 9 HUN-REN-SU Translational Extracellular Vesicle Research Group, Budapest, Hungary; 10 HCEMM-SU Extracellular Vesicle Research Group, Budapest, Hungary; 11 Department of Biostatistics, Fielding School of Public Health, University of California Los Angeles, Los Angeles, CA 90095 USA; 12 San Diego Institute of Science, Altos Labs, San Diego, CA 92121 USA; 13 Faculty of Sport Sciences, Waseda University, Tokorozawa, 2-579-15 Japan

**Keywords:** Aging clock, DNAmFitAge, Epigenetics, Extracellular vesicles, Physical exercise, Proteomics

## Abstract

Extracellular vesicles (EVs) are implicated in inter-organ communication, which becomes particularly relevant during aging and exercise. DNA methylation-based aging clocks reflect lifestyle and environmental factors, while regular exercise is known to induce adaptive responses, including epigenetic adaptations. Twenty individuals with High-fitness (aged 57.7 ± 9.8 years) and twenty Medium–Low-fitness (aged 57.5 ± 9.7 years) subjects provided blood samples. EVs were isolated from the samples using a size exclusion chromatography (SEC)-based method, and their protein content was analyzed by mass spectrometry (MS). Acceleration of the biological age estimator DNAmFitAge (AgeAccelFit) was associated with the protein cargo of EVs, whereas PhenoAge and GrimAge acceleration did not show a significant relationship. This finding suggests that the epigenetic aging-modulating role of exercise may involve inter-organ communication via EVs. Set Enrichment Analysis was performed to identify enriched Gene Ontology (GO) terms for sets of proteins that were either correlated with AgeAccelFit or detected exclusively in individuals with high levels of aerobic fitness. The protein cargo of EVs further suggests that inter-organ communication influences inflammation, the immune system, cellular repair, adhesion, metabolism and coagulation. Our findings help to understand the preventive role of exercise, which could be mediated in part by EVs.

**Supplementary Information:**

The online version contains supplementary material available at 10.1007/s10522-024-10177-9.

## Introduction

Aging is a complex process that varies among individuals. Environmental factors and lifestyle choices play a significant role in influencing the rate of aging, which can be assessed using DNA methylation-based epigenetic clocks (Lu et al. 2022). These epigenetic aging clocks are predictive of mortality risk and are closely associated with various clinical biomarkers. (Lu et al. 2019). Recently, we developed a new aging clock, DNAmFitAge, which serves as a biological age indicator by integrating measures of physical fitness, such as VO_2_max and grip strength. (McGreevy et al. 2023). Physically active individuals tend to have a younger DNAmFitAge and better age-related outcomes, including a lower mortality risk, reduced risk of coronary heart disease, and improved disease-free status. (Jokai et al. 2023).

Physical exercise, which is a potential modulator of the aging process, increases the blood flow and the metabolism by many folds in the heart, working skeletal muscle and to some extent in the brain. In contrast, it decreases blood flow significantly in the liver, kidney, and gut, having beneficial systemic effects on the whole body (Radak et al. 2008). It is highly likely that inter-organ communication is mediated partially by extracellular vesicles (EVs). EVs are a diverse group of membrane-enclosed particles present in various cell types throughout the body. They transport many biological molecules, including proteins, nucleic acids, lipids, glycans and metabolites, delivering them to other cells and tissues. EVs are classified into two main categories based on their formation: exosomes and ectosomes. However, given that most studies do not provide direct evidence for the biogenesis of the studied EVs, it is recommended to classify them using operational terms like their biophysical properties or cellular source. (Upadhya & Shetty 2024). Their capacity to reflect the state of the releasing cells and modulate the functions and phenotypes of recipient ones underscores their substantial potential as biomarkers and therapeutic agents. This significant promise has spurred considerable interest, as evidenced by the growing number of scientific publications dedicated to EV research. (van Niel et al. 2018; Buzas 2023; Sheta et al. 2023; Welsh et al. 2024).

Indeed, a growing body of evidence shows that during physical exercise, the level of EVs in circulation is increased. **(**Llorente et al. 2024). Moreover, it is suggested that exercise-induced EVs could contribute to the well-established preventive effects of exercise in a variety of diseases, ranging from cancer and fatty liver disease to Alzheimer's disease. (Zhang et al. 2022; Georgieva et al. 2023; Llorente et al. 2024). It has been reported that skeletal muscle (Huang et al. 2024), gut microbiome (Zhang et al. 2022), brain (Delgado-Peraza et al. 2023), liver (Lou et al. 2022), heart, kidney, and adipose tissue (Estébanez et al. 2020) could be the origin of circulating EVs during exercise.

It has been shown that higher levels of physical fitness are associated with a decreased incidence of diseases and a slower rate of aging. (Radak et al. 2024a). Data suggest that exercise-induced EVs associated with inter-organ communication could be involved in this correlation (Lou et al. 2022; McIlvenna & Whitham 2023; Wang et al. 2023; Delgado-Peraza et al. 2023; Radak et al. 2024a). It is known that lifestyle and environmental factors can significantly alter the pace of aging, which can be assessed by DNA-methylation-based aging clocks (Hannum et al. 2013; Horvath 2013). Here, we suggest that EVs may serve as important messengers of inter-organ communication, influencing the rate of aging as assessed by DNA methylation-based aging clocks.

## Materials and methods

### Participants

Ethical approval was obtained from the Hungarian Ministry of Human Capacities (EMMI) in agreement with the Hungarian Scientific and Research Ethics Committee (ETT TUKEB, 25,167–6/2019 EUIG). Volunteers were enrolled in the study only if they did not meet any of the following exclusion criteria: cardiovascular or musculoskeletal disorders preventing physical exertion; acute or chronic conditions affecting the kidneys, liver, or gastrointestinal system; infectious diseases, including febrile conditions; metabolic or endocrine disorders; bleeding disorders or coagulation abnormalities; cancer; autoimmune diseases; asthma; psychological or mental illnesses; limited physical or communication abilities; chronic alcohol consumption; or a history of regular smoking. Subjects were informed about the study procedures and provided written consent to participate. Prior to blood collection and fitness tests, participants completed a questionnaire detailing their exercise history, exercise frequency, and basic dietary patterns.

High- and Medium–Low-fitness categories were established using age group-stratified normative data (Kaminsky et al. 2015; Jokai et al. 2023). Individuals with VO_2_max estimates above the 75th percentile for their age group were classified as High-fitness, while those below this threshold were classified as Medium–Low-fitness. In our research, we similarly formed two groups: a High-fitness group (High-fit) and a Medium–Low-fitness group (Med-Low-fit), representing distinct age-adjusted levels of cardiorespiratory fitness. A total of forty female volunteers were classified based on their estimated VO_2_max. Data collection took place on two occasions: at the 2019 World Rowing Masters Regatta in Velence, Hungary, and at the Hungarian University of Sport Sciences in Budapest, Hungary, under similar conditions. Data and biological samples were collected at seven stations, where physiological tests were performed, participant consent was obtained, samples were collected, and medical histories were documented. We instructed the participants to have their last training session at least 24 h prior to the blood test. Of the participants, twenty were classified as Med-Low-fit (mean age ± SD = 57.5 ± 9.7), and twenty as High-fit (mean age ± SD = 57.7 ± 9.8). The participants in this study were volunteers. VO_2_max was used as the basis for participant grouping because it is widely regarded as the gold standard for assessing cardiorespiratory fitness. Low VO_2_max levels are strongly associated with an increased risk of cardiovascular disease and all-cause mortality (Ross et al. 2016), making it a critical indicator of physical health and a potential key contributor to the systemic benefits of exercise.

### Physical fitness and verbal short-term memory assessment

Physical fitness and verbal short-term memory were assessed using several key markers. Maximal grip strength was assessed with a hand dynamometer (Camry EH 101, USA) in three trials using the dominant hand, with the highest value (GripMax) recorded and normalized by body weight. Explosive lower body strength was measured by maximal vertical jump height using a linear encoder system (Muscle Lab, Norway). VO_2_max, an indicator of relative aerobic capacity, was estimated using the Chester step test (Buckley et al. 2004). Additionally, verbal short-term memory was evaluated through the digit span test (Choi et al. 2014).

### Blood based biomarkers

Blood collection and processing details were discussed earlier (Jokai et al. 2023). Blood was drawn from the right cubital vein into EDTA tubes for DNA cytosine methylation analysis, and serum samples were collected for LDL and HDL measurement. DNA was extracted from whole blood and bisulfite-converted using the EZ-96 DNA Methylation MagPrep Kit (Zymo Research, Irvine, CA, USA). Genome-wide DNA methylation was assessed with the Infinium MethylationEPIC BeadChip following (Illumina Inc., San Diego, CA) standard protocols. DNA methylation-based biological age estimators, including DNAmFitAge, PhenoAge, and GrimAge accelerations, were calculated as described by McGreevy et al. (2023), and Lu et al. (2019), respectively. The LDL and HDL parameters were assessed using standard clinical chemistry techniques.

### Preparation of platelet-free plasma (PFP) from whole blood

Qualified nurses collected venous blood samples (16 mL) from the cubital vein of subjects using two anticoagulant citrate dextrose-A (ACD-A) tubes via the BD Vacutainer blood collection system. The tubes were centrifuged at 2,500 × g for 15 min using an Eppendorf 5804R centrifuge (Eppendorf AG, Hamburg, Germany). The resulting supernatant, which was platelet-poor plasma (PPP), was transferred into a 5 mL Eppendorf tube and centrifuged again at 2500 × g for 15 min (Lacroix et al. 2012). The final supernatant comprising PFP, was aliquoted into microcentrifuge tubes, frozen on dry ice, and stored at − 80 °C until use.

### Pre-Analytical assessment of platelet-free plasma (PFP)

Pre-analytical assessments were performed to evaluate the quality of the PFP and identify any factors potentially interfering with the isolation process (Fliser et al. 2012; Welsh et al. 2024). Platelet and haemoglobin levels were measured using a Sysmex XE2100 haematology analyser (Sysmex Corporation, Kobe, Japan), while absorbance was measured at different wavelengths with LabSystems Multiskan ELISA reader (Thermo/LabSystems, Vantaa, Finland). The results can be found in Supplementary Validation S1. The precise description and exclusion criteria were derived from our previous study by György et al. (2024).

### Isolation of small extracellular vesicles (sEVs)

Each experimental procedure commenced with the isolation of sEVs from 2.5 mL of PFP. Analysis of the isolated sEVs included measurements using a Nanodrop spectrophotometer, nanoparticle tracking analysis (NTA), transmission electron microscopy (TEM), and mass spectrometry (MS).

Frozen samples were thawed at room temperature (RT) for approximately 1 h. PFP samples (2.5 mL) were diluted to 5 mL with 0.2–0.1 µm tandem filtered NaCl-Hepes buffer and filtered through a 0.8 µm sterile cellulose acetate (CA) syringe filter to remove larger particles (György et al. 2014; Gaspar et al. 2020). The filtered PFP was then centrifuged at 18,000 × g for 20 min to obtain a supernatant rich in sEVs. It was further filtered through a 0.2 µm CA syringe filter and concentrated using a 100 kDa (Amicon Ultra-4 Centrifugal Filter Unit; Merck, New York, NY, USA) ultrafiltration tube (3000 × g for 30 min; 5804R Eppendorf, Eppendorf AG, Hamburg, Germany). Samples were then adjusted to 1500 µL with buffer and centrifuged at 10,000 × g for 10 min (Z216MK Hermle centrifuge, HermLe Labortechnik GmbH, Wehingen, Germany) to remove aggregates. We employed size exclusion chromatography (SEC) (70 nm qEV-original column; IZON Science, Cambridge, MA, USA) to isolate vesicles, aiming to obtain a fraction enriched in sEVs while minimizing contamination by protein and lipoprotein particles (Brennan et al. 2020). Samples (1500 µL) were loaded onto the SEC column using NaCl-HEPES buffer for elution. Fractions were collected as follows: waste fraction (3 mL), followed by fractions 1–4 (0.5 mL each). The pooled fractions containing sEVs from fractions 1–4 were transferred to a UC centrifuge tube (#:344,619; Beckman Coulter, Brea, CA, USA), filled with NaCl-Hepes buffer, and centrifuged at 100,000 × g for 60 min (Type-100, Beckman Coulter centrifuge). After removing the supernatant, the resulting pellet containing sEVs was resuspended in 15–20 µL of NaCl-Hepes buffer and stored at − 80 °C until subsequent MS analysis.

The methodology employed for sEVs in this study follows our previously published work (György et al. 2024), which includes our detailed step-by-step isolation protocol.

### Spectrophotometry

The total protein content was quantified using a NanoDrop ND-1000 instrument manufactured by Thermo Fisher Scientific (Waltham, MA, USA). Absorbance was read at 280 nm, with NaCl-Hepes solution used as the reference (1.5 µL/sample, repeated 3 times).

### Nanoparticle tracking analysis (NTA)

Particle size distribution and concentration were assessed using a ZetaView Z-Nanoparticle Tracking Analysis instrument (Particle Metrix GmbH, Inning am Ammersee, Germany). It should be noted that this method does not distinguish between "vesicular" and "non-vesicular" particles such as protein complexes and aggregates (Filipe et al. 2010). Prior to ultracentrifugation (UC), the size distribution and concentration of sEVs from samples were determined using various dilutions (100-200x). Sample settings included auto exposure, gain: 28.8, offset: 0, shutter: 100, and sensitivity: 80. Data analysis was performed using ZetaView Analysis software 8.05.10, utilizing a minimum of 8 positions for each analysis.

### Transmission electron microscopy (TEM)

Whole-mounted sEVs were visualized using the protocol by Théry et al. (2018), with adaptations of established immunogold TEM techniques (Théry et al. 2006; Koncz et al. 2023). For EV visualization, 5 µL of sample was placed on formvar-coated nickel grids (SPI Supplies, West Chester, PA, USA) and incubated for 10 min at RT. Excess liquid was removed, and EVs were fixed with 4% paraformaldehyde for 10 min at RT, followed by three washes with distilled water. A 2% sucrose/PBS (Molar Chemicals, Halásztelek, Hungary) blocking solution was then applied and incubated for 1 h at RT to reduce nonspecific binding (Koncz et al. 2023).

Primary antibodies (rabbit anti-CD9 IgG antibody; Abcam, Cambridge, UK) were applied overnight at 4 °C in 2% sucrose/PBS. After three 5-min washes with 2% sucrose/PBS at RT, secondary antibodies (polyclonal goat anti-rabbit IgG conjugated with 5 nm gold particles; Sigma, Darmstadt, Germany) were applied for 1 h in the same solution. The choice of 2% sucrose helped to reduce nonspecific binding and immunoreactivity to BSA. Following secondary antibody application, samples underwent three 5-min RT washes and PBS rinses (3 times, 5 min each). Samples were post-fixed with 2% glutaraldehyde, followed by three 5-min washes with distilled water at RT (Koncz et al. 2023). Immunogold-labelled samples were subjected to positive–negative contrasting, and were examined using a JEOL 1011 transmission electron microscope (Tokyo, Japan). Detailed antibody information can be found in Supplementary Table S1.

### Mass spectrometry (MS)

Isolated vesicle samples were subjected to freeze–thaw cycles (Turiák et al. 2011) and then the proteins were precipitated with 9 × volume ice cold ethanol overnight at -20 °C. The pellet was washed twice and then re-dissolved in 20 µL 8 M urea in 50 mM ammonium-bicarbonate. The proteins were reduced with 5 mM DTT at 37 °C for 30 min, then samples were alkylated with 10 mM iodoacetamide at RT in the dark for 30 min. Next, samples were diluted tenfold with 50 mM ammonium bicarbonate and in-solution digestion was performed first with Lys-C/trypsin mixture at 1:100 weight ratio at 37 °C for 1 h, then with trypsin at 1:10 weight ratio at 37 °C overnight. Digestion was stopped by adding 1 µL formic acid, then samples were desalted on Pierce C18 spin columns (György et al. 2024). The samples were dried down in a vacuum concentrator, re-dissolved in 30 µL 0.1% FA/3% ACN, then 15 µL of the samples was injected into an UltiMate 3000 RSLCnano HPLC (Thermo Fisher Scientific) coupled to an Orbitrap Exploris 240 (Thermo Fisher Scientific) mass spectrometer for LC–MS/MS analysis. HPLC and MS acquisition parameters can be found in Supplementary Validation S3.

Peptides and proteins were searched against the UniProt human database (access date: 03/2024) using Byonic (Bern et al. 2013). Mass tolerance was set to 20 ppm for precursor and fragment ions, allowing 2 missed cleavages. Carbamidomethylation was set as a fixed modification, while modifications suggested by Byonic Preview (oxidation, deamidation, pyro-Glu formation, ammonia loss, and acetylation) were variable. Identified proteins were combined into a fasta file and used for protein quantification with MaxQuant 1.6.17.0. (Tyanova et al. 2016). Quantification was performed using the LFQ algorithm, with oxidation and deamidation as variable modifications and a peptide mass tolerance of 10 ppm. Contaminant proteins, reverse sequences, and proteins quantified by a single peptide were excluded from analysis.

### Statistical analysis

Statistical analysis of sEVs was performed using GraphPad Prism 9.4.1. ANOVA was conducted to compare variances between group means, while associations between physical fitness markers and protein content were assessed using Pearson's correlation, implemented in R version 4.4.1 with the nortest, lmtest, and WGCNA packages. (Langfelder & Horvath 2008). Homoscedasticity and normality were assessed using the Breusch-Pagan test and the Lilliefors test, respectively. If either assumption was violated, Spearman's correlation was used instead of Pearson's correlation. To control for multiple testing errors, q-values were calculated and FDR was set at 0.05. The raw LFQ values were first log2 transformed to approximate a normal distribution, and these transformed values were used in subsequent analyses. Only proteins present in at least 90% of the samples were included in the correlation analysis.

Protein set enrichment analysis (PSEA) was performed using the PANTHER (protein analysis through evolutionary relationships) classification framework (Mi et al. 2013). UniProt IDs were submitted to the Gene Ontology (geneontology.org) online interface (Aleksander et al. 2023) using the PANTHER overrepresentation test (v20240807) as the analysis type. The annotation version and release date were DOI: 10.5281/zenodo.12173881 and 2024–06-17, respectively. The reference list was set to Homo sapiens (n = 20,580). The analyses were conducted across three main gene ontology domains—cellular component (CC, GO:0005575), biological process (BP, GO:0008150), and molecular function (MF, GO:0003674)—as well as PANTHER protein classes (version 19.0). Overrepresentation was tested by Fisher’s exact test and with FDR correction.

## Results

**Fig. 1:**
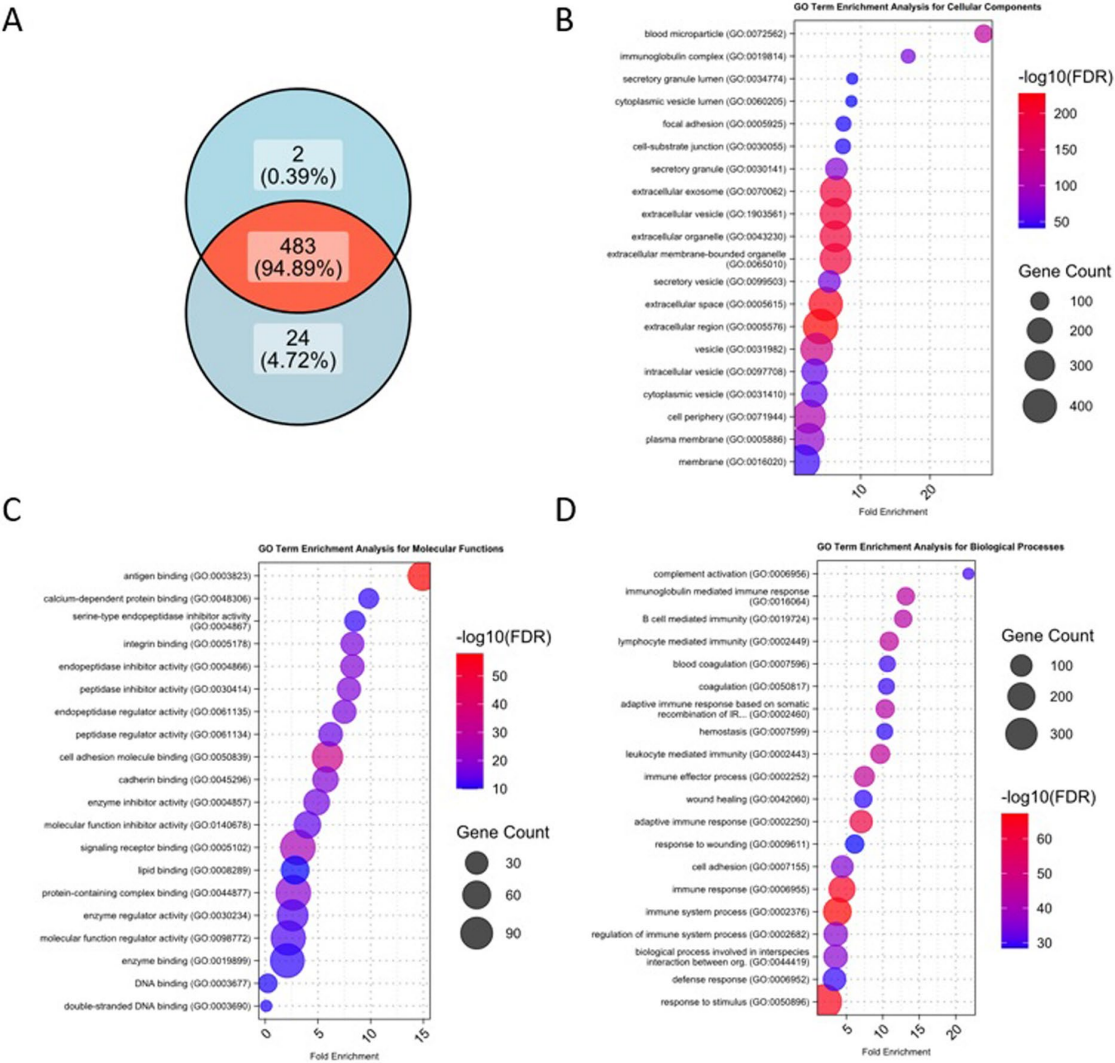
Characteristics of the detected vesicular proteins. A total of 509 distinct proteins were identified. (**A**) illustrates their presence across the Med-Low- and High-fit groups. Figure displays the top 20 Gene Ontology (GO) term enrichment results in the main categories: (**B**) Cellular Component, (**C**) Molecular Functions and (**D**) Biological Processes

Proper isolation of EVs is fundamental to obtain accurate, reliable, and reproducible results, which are essential for both quality research and clinical applications. Figure 1, and Supplementary Fig. S1 show the characterization of EVs and the protein contents obtained in the present study. The detailed results of the analysis can be read in Supplementary Validation S1 and S2.

**Fig. 2:**
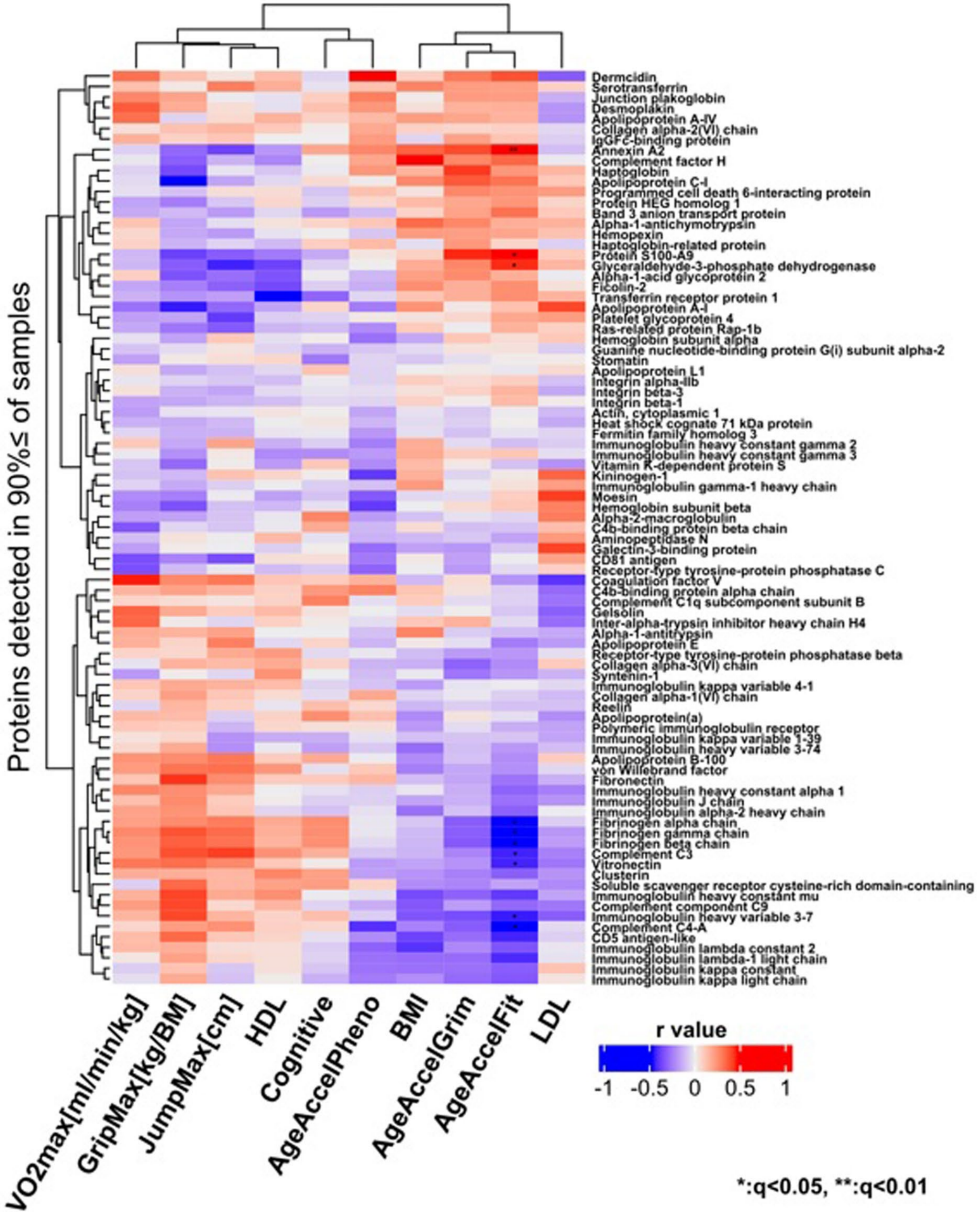
Heat map depicting the correlation between protein mass spectrometry normalized intensity variables and physiological markers. These markers include epigenetic aging (epigenetic acceleration measured by GrimAge-Acceleration, PhenoAge-Acceleration, and DNAmFitAge-Acceleration, physical fitness (relative aerobic capacity: VO_2_max [ml/kg/min], relative maximal grip strength: GripMax [kg/BM], maximal jump height: JumpMax [cm]), clinical markers (body mass index: BMI, low-density lipoprotein: LDL, high-density lipoprotein: HDL), and cognitive performance (digit span test)

A heat map was generated to visualize the correlation between normalized protein mass spectrometry intensity variables and physiological markers (Fig. 2). There was no difference in protein abundance between High- and Med-Low-fit individuals, similarly no significant associations were detected between protein levels and physiological fitness indicators at FDR < 0.05 level. In our sample, no correlation between memory function (cognitive), blood lipid markers (LDL, HDL), and BMI reached statistical significance after correction for multiple comparisons.

**Fig. 3:**
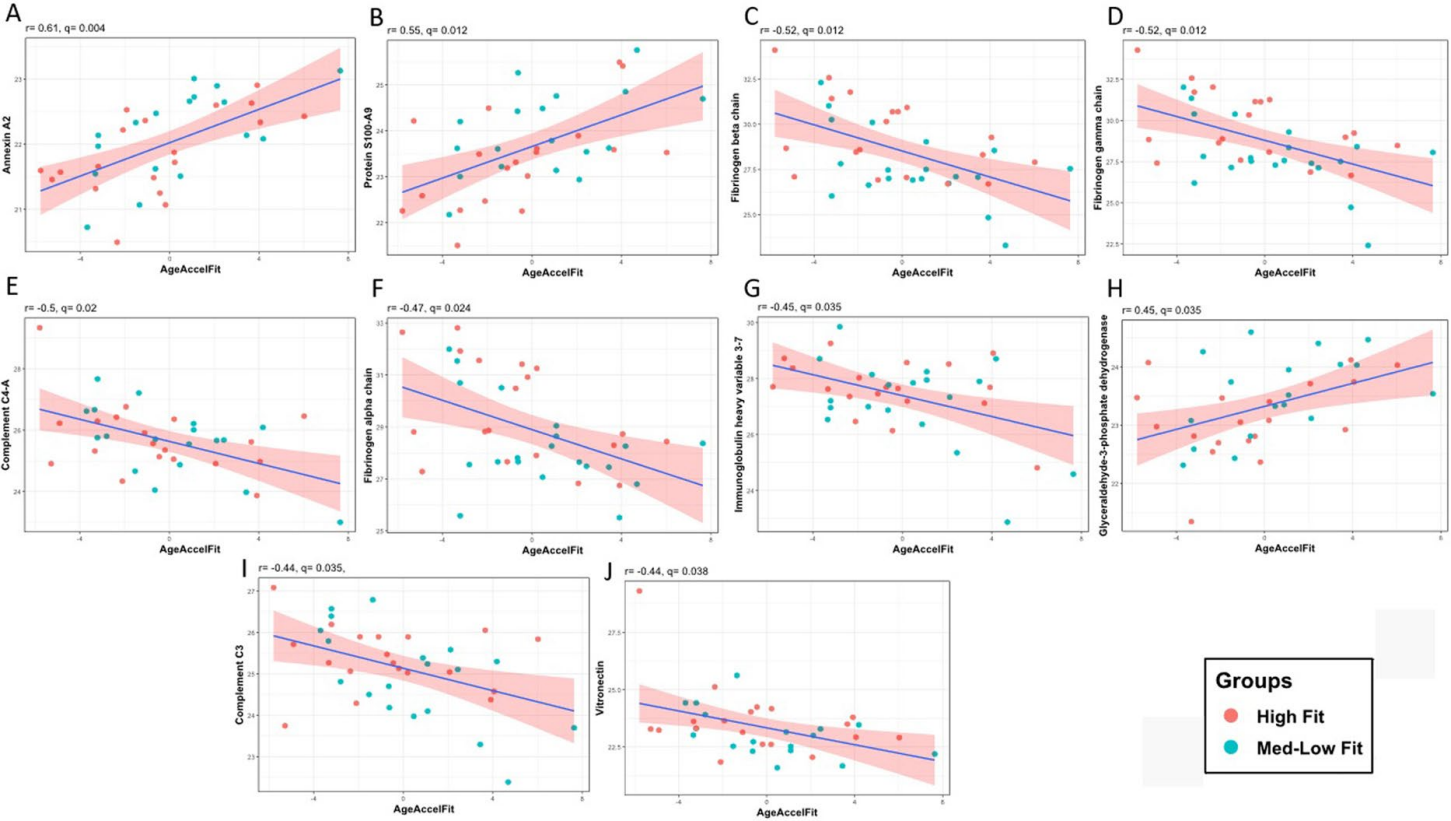
Association between the biological aging estimator, DNAmFitAge-Acceleration (depicted in years), and normalized log2-transformed protein abundance levels: **A** Annexin A2, **B** Protein S100-A9, **C** Fibrinogen beta chain, **D** Fibrinogen gamma chain, **E** Complement C4-A, **F** Fibrinogen alpha chain, **G** Immunoglobulin heavy variable 3–7, **H** Glyceraldehyde-3-phosphate dehydrogenase, **I** Complement C3, **J** Vitronectin

The age acceleration of the three aging clocks, PhenoAge, GrimAge, and DNAmFitAge was evaluated in a relationship with the cargo of EVs. According to our findings, 10 proteins are associated with DNAmFitAge-Acceleration at a q < 0.05 level (Fig. 3). Significant associations were found between Annexin 2A, (r = 0.61, q = 0.004, n = 36), Protein S100-A9 (r = 0.55, q = 0.012, n = 37), Fibrinogen beta chain (r = -0.52, q = 0.012, n = 39), Fibrinogen gamma chain (r = -0.52, q = 0.012, n = 39), Complement C4-A (r = -0.5, q = 0.02, n = 37), Fibrinogen alpha chain (r = -0.47, q = 0.024, n = 39), Immunoglobulin heavy variable 3–7 (r = -0.45, q = 0.035, n = 38), Glyceraldehyde-3-phosphate dehydrogenase (r = 0.45, q = 0.035, n = 37), Complement C3 (r = -0.44, q = 0.035, n = 38), Vitronectin (r = -0.44, q = 0.038, n = 37).

Other biological age indicators, such as GrimAge-Acceleration and PhenoAge-Acceleration, showed no significant correlation with MS data.

In case of protein set enrichment, three sets of conditions were analyzed: (i) all proteins detected by mass spectrometry (n = 509) to verify vesicular origin, (ii) proteins present only in the High-fit classified subjects (n = 24) (Fig. 1A), and (iii) proteins showing significant association with FitAgeAcceleration at the FDR < 0.05 level (n = 10). For the enrichment of all detected proteins, Fig. 1B-D indicates showed number of highly significant results that are indicating microvesicular origin including: Blood microparticle (GO:0072562), extracellular exosome and vesicle (GO:0070062, GO: 1,903,561).

**Fig. 4:**
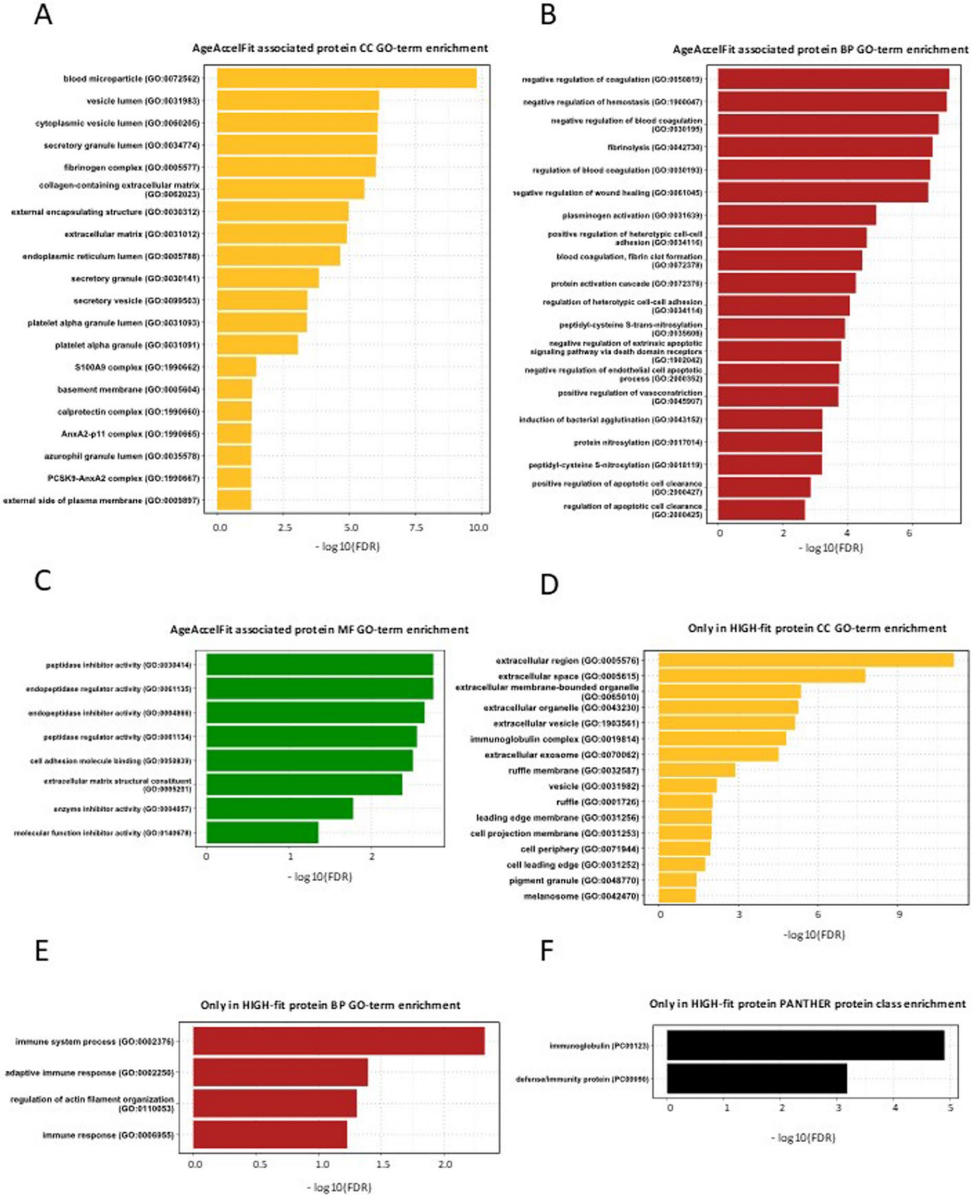
Enrichment analysis of proteins associated with DNAmFitAge-Acceleration (**A, B, C**). CC: gene ontology term cellular component, BP: gene ontology term biological processes, MF: gene ontology term molecular function. Enrichment analysis of proteins detected exclusively in High-fit (**D, E, F**) individuals. PANTHER: Protein analysis through evolutionary relationships

We performed GO pathway to estimate the nature of only in High-fit and DNAmFitAge-Acceleration associated proteins from vesicle samples (Fig. 4). Gene Ontology (GO) enrichment analysis revealed significant enrichment of proteins across biological processes, cellular components, molecular functions, and PANTHER protein classes. However, for AgeAccelFit, no enrichment was observed in the PANTHER protein class. Similarly, the protein set detected exclusively in the High-Fit group did not show enrichment for molecular function GO terms. The proteins were significantly enriched in the ‘extracellular region’, ‘extracellular space’, and ‘blood microparticle’ in the cellular component. Meanwhile, significant enrichments of proteins involved in the ‘immune system process’, ‘adaptive immune response’, coagulation, and hemostasis were observed in biological processes. Based on our further observations, many proteins were associated with protease regulation and cell adhesion in molecular function analysis. Table 1 shows 24 proteins that were present only in one or more High-fit classified subjects.

**Table 1:** Proteins that were present only in one or more High-fit classified subjects

Protein name	n (equals the number of positive detections in their respective group)
Alpha-2-antiplasmin	9
Immunoglobulin lambda variable 9–49	5
Immunoglobulin lambda variable 4–60	4
SH3 domain-binding glutamic acid-rich-like protein 3	4
Immunoglobulin heavy variable 3–73	3
Immunoglobulin heavy variable 4–4	3
SH3 and multiple ankyrin repeat domains protein 1	3
A disintegrin and metalloproteinase with thrombospondin motifs 13	2
Amino acid transporter heavy chain SLC3A2	2
Apolipoprotein A-V	2
Carboxypeptidase N catalytic chain	2
Cartilage acidic protein 1	2
Cathelicidin antimicrobial peptide	2
Complement C2	2
Epidermal growth factor receptor kinase substrate 8	2
Growth factor receptor-bound protein 2	2
Immunoglobulin heavy variable 3–53	2
Immunoglobulin kappa variable 1D-8	2
Inter-alpha-trypsin inhibitor heavy chain H3	2
Plastin-2	2
Pleckstrin	2
Protein disulfide-isomerase	2
Ras-related protein Rab-35	2
Synaptic vesicle membrane protein VAT-1 homolog	2

In case of Med-Low-fit only 2 proteins are detected only in one more Med-Low-fit volunteer: Transforming growth factor beta activator LRRC32 (n = 2), Equilibrative nucleoside transporter 1 (n = 2).

Interestingly, there was no association with the number of years spent in regular exercise or with basic dietary habits (Supplementary Fig. S2). On the other hand, weekly hours spent exercising were positively correlated with Coagulation Factor V (rho = 0.75, q = 0.0002), C4b Binding Protein Alpha Chain (rho = 0.7, q = 0.0007), and Complement C1q Subcomponent Subunit B (rho = 0.67, q = 0.0027).

## Discussion

Aging is a complex process that occurs in an organ-specific and individual manner. In this study, we identified the protein cargo of EVs related to DNA methylation-based aging processes, specifically PhenoAge, GrimAge, and DNAmFitAge. Previous research has shown that both PhenoAge and GrimAge are excellent predictors of mortality. (Horvath & Raj 2018). It turns out that DNAmFitAge, which incorporates physiological function-related DNA methylation sites (McGreevy et al. 2023), is the only aging clock associated with the proteomics of EVs. This raises the possibility that exercise-produced EVs are important players in the progress of epigenetic aging.

There is growing evidence that higher levels of physical fitness are associated with a decreased incidence of diseases, increased longevity (Lang et al. 2024), and a slower rate of aging (Sánchez-Sánchez et al. 2024). Recent reports indicate that Olympic champions experience a decelerated aging process compared to recreational athletes and sedentary individuals (Radak et al. 2024b). The molecular mechanisms underlying the beneficial effects of exercise on aging are highly complex. These include the maintenance of telomere length (Seki et al. 2023), upregulation of “anti-aging” proteins such as klotho and sirtuins (Aczel et al. 2023; Koltai et al. 2009), enhanced protection against oxidative stress (Radak et al. 2008), rejuvenation of the mitochondrial network (Koltai et al. 2012), and upregulation of the immune system (Simpson et al. 2012), to name a few. DNA methylation-based aging clocks aim to capture the rate of aging, and the continuous development of these clocks suggests that their evolution is still ongoing.

PhenoAge and GrimAge are remarkable mortality predictors. Aging is associated with a decline in most physiological functions, including strength and endurance, both of which are linked to the prevention of a wide range of diseases and decreased mortality (Allen et al. 2017). However, due to the limited sensitivity of PhenoAge and GrimAge to these factors, our group developed DNAmFitAge, which incorporates genes related to physiological functions (McGreevy et al. 2023). The exclusive correlation between the protein content of EVs and DNAmFitAge further supports the idea that physical fitness levels modulate the rate of aging and that EVs serve as important inter-organ messengers of epigenetic aging.

It has been proposed that EVs can be involved in the prevention of many life-style dependent diseases. The protein cargo of EVs helps, at least in part, to understand some mosaics of this preventive effects.

Annexin A2 is a multifunctional calcium- and phospholipid-binding protein. Higher levels of Annexin A2 in EVs, which correlate with DNAmFitAge acceleration, could enhance cellular uptake of EVs, thereby contributing to processes such as angiogenesis. (Desai et al. 2022). Moreover, Annexin A2 can modulate inflammation and, similar to protein S100-A9, which also positively correlates with DNAmFitAge acceleration, can act as either a foe or a friend in cellular processes, depending on the microenvironment. The presence of fibrinogen beta in EVs, which negatively correlates with DNAmFitAge acceleration, serves as a biomarker for cardiovascular diseases and cancer. (Liu et al. 2024). Additionally, the fibrinogen content of EVs can be involved in thrombus formation (Suades et al. 2022). Complement C4A and C3 in EVs can be an important regulator of immune response and inflammation, and communication. Immunoglobulin cargo of EVs is also involved and immune and metabolic response (Nederveen et al. 2021). The function of glyceraldehyde-3-phosphate dehydrogenase (GAPDH) in EVs, may include suppression of inflammation via TNF-alfa (Das et al. 2021), GAPDH cargo can promote extensive EV clustering, enhance the therapeutic potential of EVs in the brain (Dar et al. 2021) and regulate metabolism (Romani et al. 2022). Vitronectin is a glycoprotein that plays a significant role in various physiological processes, including cell adhesion, migration, and tissue repair. Additionally, vitronectin in EVs is involved in bone density. (Pepe et al. 2022).

The proteomics of EVs in High-fit and Med-Low-fit groups showed some differences. Besides the above-mentioned proteins, alpha-2-antiplasmin (A2AP), which is a key inhibitor of fibrinolysis, was present in the EVs of nine subjects from the 20 in High-fit but not in Med-Low-fit group. Alpha-2-antiplasmin as a cargo of EVs can influence the balance between coagulation and fibrinolysis (Singh et al. 2024), which is crucial during intense physical activity to prevent excessive bleeding or clotting. SH3BGRL3 was present in EVs of four High-fit but not in Med-Low-fit subject is one of the proteins found in EVs believed to be involved in modulating cellular responses to the physical stress of exercise (Di Pisa et al. 2021). Moreover, at a lower level of the organism SH3BGRL3 is involved in redox regulation (Mazzocco et al. 2001).

Weekly hours of exercise training is associated with proteins are linked to key biological processes such as coagulation, immune response, and complement activation The positive correlation between EV cargo C4b Binding Protein Alpha Chain (C4BPA) levels and weekly hours spent exercising observed in our study may reflect the potential of regular physical activity to modulate immune functions. This aligns with findings from recent research demonstrating that C4BPA plays a critical role in promoting the accumulation of CD8 + tumor-infiltrating lymphocytes and enhancing the tumor microenvironment to counteract cancer progression (Sasaki et al. 2021). Such evidence highlights the broader systemic benefits of exercise, not only on general health but also on immune-related pathways.

A few studies have measured the protein cargo of EVs in healthy-aged subjects. A recent study by Chong et al. (2024) examined the proteomics of EVs after an acute 20-min cycling exercise at 70% of estimated VO2peak. The protein content of EVs is related to both physical fitness levels and age. Despite differences in protocols, we found some overlap in the protein cargo with the findings of this study.

This study has many limitations. We used only female subjects because we had volunteers who participated in a Master Rowing Championship. We excluded volunteers whose blood lipid profiles could significantly affect the quality of EV isolation. The variation in ages, training histories, levels of physical fitness, lifestyles, and nutritional habits (which could not be standardized prior to the competition) may have influenced our results. However, despite these limitations, we strongly believe that the current study provides valuable novel insights into the involvement of EVs in lifestyle-related aging. We are investigating organ-dependent DNA methylation in aging and predict that identifying the donors and acceptors of EVs involved in exercise-induced adaptations will be a crucial step toward a deeper understanding of the systemic effects of exercise on aging.

In summary, the proteomics cargo of EVs obtained from High-fit and Med-Low-fit subjects correlated with DNAmFitAge acceleration strongly suggesting that epigenetic aging modulating role of exercise involves inter-organ communication via EVs. The characteristics of the protein cargo of EVs further indicate that the inter-organ communication influences inflammation, the immune system, cellular repair, adhesion, metabolism and coagulation. It is suggested that the EVs-associated modulation of DNA methylation-based aging is related to the level of physical fitness. Our findings help to understand the preventive role of exercise, which is in part, could be mediated by EVs.

## Supplementary Information

Below is the link to the electronic supplementary material.

Supplementary file1 (PDF 208 KB)

Supplementary file2 (PDF 421 KB)

Supplementary file3 (DOCX 21 KB)

Supplementary file4 (DOCX 16 KB)

## Data Availability

The datasets generated during and/or analyzed during the current study are available from the corresponding author on reasonable request.

## Abbreviations

**ANOVA**: Analysis of variance
**EVs**: Extracellular vesicles
**GO**: Gene ontology
**MS**: Mass spectrometry
**NTA**: Nanoparticle tracking analysis
**UC**: Ultracentrifugation
**PBS**: Phosphate-buffered saline
**PFP**: Platelet-free plasma
**PPP**: Platelet-poor plasma
**RT**: Room temperature
**SEC**: Size exclusion chromatography
**TEM**: Transmission electron microscopy

## References

[CR1] Aczel D, Torma F, Jokai M, McGreevy K, Boros A, Seki Y, Boldogh I, Horvath S, Radak Z (2023) The circulating level of klotho is not dependent upon physical fitness and age-associated methylation increases at the promoter region of the klotho gene. Genes (Basel) 14(2):525. 10.3390/genes14020525 36833453 10.3390/genes14020525 PMC9957177

[CR2] Aleksander SA, Balhoff J, Carbon S, Cherry JM, Drabkin HJ, Ebert D, Feuermann M, Gaudet P, Harris NL, Hill DP, Lee R, Mi H, Moxon S, Mungall CJ, Muruganugan A, Mushayahama T, Sternberg PW, Thomas PD, Van Auken K, Ramsey J, Siegele DA, Chisholm RL, Fey P, Aspromonte MC, Nugnes MV, Quaglia F, Tosatto S, Giglio M, Nadendla S, Antonazzo G, Attrill H, Dos Santos G, Marygold S, Strelets V, Tabone CJ, Thurmond J, Zhou P, Ahmed SH, Asanitthong P, Luna Buitrago D, Erdol MN, Gage MC, Ali Kadhum M, Li KYC, Long M, Michalak A, Pesala A, Pritazahra A, Saverimuttu SCC, Su R, Thurlow KE, Lovering RC, Logie C, Oliferenko S, Blake J, Christie K, Corbani L, Dolan ME, Drabkin HJ, Hill DP, Ni L, Sitnikov D, Smith C, Cuzick A, Seager J, Cooper L, Elser J, Jaiswal P, Gupta P, Jaiswal P, Naithani S, Lera-Ramirez M, Rutherford K, Wood V, De Pons JL, Dwinell MR, Hayman GT, Kaldunski ML, Kwitek AE, Laulederkind SJF, Tutaj MA, Vedi M, Wang SJ, D’Eustachio P, Aimo L, Axelsen K, Bridge A, Hyka-Nouspikel N, Morgat A, Aleksander SA, Cherry JM, Engel SR, Karra K, Miyasato SR, Nash RS, Skrzypek MS, Weng S, Wong ED, Bakker E, Berardini TZ, Reiser L, Auchincloss A, Axelsen K, Argoud-Puy G, Blatter MC, Boutet E, Breuza L, Bridge A, Casals-Casas C, Coudert E, Estreicher A, Livia Famiglietti M, Feuermann M, Gos A, Gruaz-Gumowski N, Hulo C, Hyka-Nouspikel N, Jungo F, Le Mercier P, Lieberherr D, Masson P, Morgat A, Pedruzzi I, Pourcel L, Poux S, Rivoire C, Sundaram S, Bateman A, Bowler-Barnett E, Bye-A-Jee H, Denny P, Ignatchenko A, Ishtiaq R, Lock A, Lussi Y, Magrane M, Martin MJ, Orchard S, Raposo P, Speretta E, Tyagi N, Warner K, Zaru R, Diehl AD, Lee R, Chan J, Diamantakis S, Raciti D, Zarowiecki M, Fisher M, James-Zorn C, Ponferrada V, Zorn A, Ramachandran S, Ruzicka L, Westerfield M (2023) The Gene Ontology knowledgebase. Genetics. 10.1093/genetics/iyad031 36866529 10.1093/genetics/iyad031 PMC10158837

[CR3] Allen L, Williams J, Townsend N, Mikkelsen B, Roberts N, Foster C, Wickramasinghe K (2017) Socioeconomic status and non-communicable disease behavioural risk factors in low-income and lower-middle-income countries: a systematic review. Lancet Glob Health 5(3):e277–e289. 10.1016/S2214-109X(17)30058-X 28193397 10.1016/S2214-109X(17)30058-X PMC5673683

[CR4] Bern M, Kil YJ, Becker C (2012) Byonic: advanced peptide and protein identification software. Current Protocols in Bioinformatics. 10.1002/0471250953.bi1320s40 23255153 10.1002/0471250953.bi1320s40 PMC3545648

[CR5] Brennan K, Martin K, FitzGerald SP, O’Sullivan J, Wu Y, Blanco A, Richardson C, Mc Gee MM (2020) A comparison of methods for the isolation and separation of extracellular vesicles from protein and lipid particles in human serum. Sci Rep 10(1):1039. 10.1038/s41598-020-57497-7 31974468 10.1038/s41598-020-57497-7 PMC6978318

[CR6] Buckley JP, Sim J, Eston RG, Hession R, Fox R (2004) Reliability and validity of measures taken during the Chester step test to predict aerobic power and to prescribe aerobic exercise. Br J Sports Med 38(2):197–205. 10.1136/bjsm.2003.005389 15039259 10.1136/bjsm.2003.005389 PMC1724781

[CR7] Buzas EI (2023) The roles of extracellular vesicles in the immune system. Nat Rev Immunol 23(4):236–250. 10.1038/s41577-022-00763-8 35927511 10.1038/s41577-022-00763-8 PMC9361922

[CR8] Choi HJ, Lee DY, Seo EH, Jo MK, Sohn BK, Choe YM, Byun MS, Kim JW, Kim SG, Yoon JC, Jhoo JH, Kim KW, Woo JI (2014) A normative study of the digit span in an educationally diverse elderly population. Psychiatry Investig 11(1):39–43. 10.4306/pi.2014.11.1.39 24605122 10.4306/pi.2014.11.1.39 PMC3942550

[CR9] Chong MC, Shah AD, Schittenhelm RB, Silva A, James PF, Wu SSX, Howitt J (2024) Acute exercise-induced release of innate immune proteins via small extracellular vesicles changes with aerobic fitness and age. Acta Physiol (Oxf) 240(3):e14095. 10.1111/apha.14095 38243724 10.1111/apha.14095

[CR10] Dar GH, Mendes CC, Kuan WL, Speciale AA, Conceição M, Görgens A, Uliyakina I, Lobo MJ, Lim WF, el Andaloussi S, Mäger I, Roberts TC, Barker RA, Goberdhan DCI, Wilson C, Wood MJA (2021) Author Correction: GAPDH controls extracellular vesicle biogenesis and enhances the therapeutic potential of EV mediated siRNA delivery to the brain. Nat Commun 12(1):7357. 10.1038/s41467-021-27700-y 34916508 10.1038/s41467-021-27700-y PMC8677793

[CR11] Das P, Mukherjee A, Adak S (2021) Glyceraldehyde-3-phosphate dehydrogenase present in extracellular vesicles from *Leishmania* major suppresses host TNF-alpha expression. J Biol Chem 297(4):101198. 10.1016/j.jbc.2021.101198 34534548 10.1016/j.jbc.2021.101198 PMC8502904

[CR12] Delgado-Peraza F, Nogueras-Ortiz C, Simonsen AH, Knight DD, Yao PJ, Goetzl EJ, Jensen CS, Høgh P, Gottrup H, Vestergaard K, Hasselbalch SG, Kapogiannis D (2023) Neuron-derived extracellular vesicles in blood reveal effects of exercise in Alzheimer’s disease. Alzheimer’s Res & Ther 15(1):156. 10.1186/s13195-023-01303-9 37730689 10.1186/s13195-023-01303-9 PMC10510190

[CR13] Desai PP, Narra K, James JD, Jones HP, Tripathi AK, Vishwanatha JK (2022) Combination of small extracellular vesicle-derived annexin A2 protein and mRNA as a potential predictive biomarker for chemotherapy responsiveness in aggressive triple-negative breast cancer. Cancers. 10.3390/cancers15010212 36612209 10.3390/cancers15010212 PMC9818227

[CR14] Di Pisa F, Pesenti E, Bono M, Mazzarello AN, Bernardi C, Lisanti MP, Renzone G, Scaloni A, Ciccone E, Fais F, Bruno S, Scartezzini P, Ghiotto F (2021) SH3BGRL3 binds to myosin 1c in a calcium dependent manner and modulates migration in the MDA-MB-231 cell line. BMC Mol Cell Biol 22(1):41. 10.1186/s12860-021-00379-1 34380438 10.1186/s12860-021-00379-1 PMC8356473

[CR15] Estébanez B, Jiménez-Pavón D, Huang CJ, Cuevas MJ, González-Gallego J (2021) Effects of exercise on exosome release and cargo in in vivo and ex vivo models: a systematic review. J Cell Physiol 236(5):3336–3353. 10.1002/jcp.30094 33037627 10.1002/jcp.30094

[CR16] Filipe V, Hawe A, Jiskoot W (2010) Critical evaluation of nanoparticle tracking analysis (NTA) by nanosight for the measurement of nanoparticles and protein aggregates. Pharm Res 27(5):796–810. 10.1007/s11095-010-0073-2 20204471 10.1007/s11095-010-0073-2 PMC2852530

[CR17] Fliser E, Jerkovic K, Vidovic T, Gorenjak M (2012) Investigation of unusual high serum indices for lipemia in clear serum samples on Siemens analysers Dimension. Biochemia Medica, . 10.11613/BM.2012.037 10.11613/bm.2012.037 PMC3900048 23092066

[CR18] Gaspar LS, Santana MM, Henriques C, Pinto MM, Ribeiro-Rodrigues TM, Girão H, Nobre RJ, Pereira de Almeida L (2020) Simple and fast SEC-based protocol to isolate human plasma-derived extracellular vesicles for transcriptional research. Mol Ther - Methods & Clin Dev 18:723–737. 10.1016/j.omtm.2020.07.012 32913880 10.1016/j.omtm.2020.07.012 PMC7452272

[CR19] Georgieva I, Tchekalarova J, Iliev D, Tzoneva R (2023) Endothelial senescence and its impact on angiogenesis in Alzheimer’s disease. Int J Mol Sci. 10.3390/ijms241411344 37511104 10.3390/ijms241411344 PMC10379128

[CR20] György B, Pálóczi K, Kovács A, Barabás E, Bekő G, Várnai K, Pállinger É, Szabó-Taylor K, Szabó TG, Kiss AA, Falus A, Buzás EI (2014) Improved circulating microparticle analysis in acid-citrate dextrose (ACD) anticoagulant tube. Thromb Res 133(2):285–292. 10.1016/j.thromres.2013.11.010 24360116 10.1016/j.thromres.2013.11.010

[CR21] György B, Pálóczi K, Balbisi M, Turiák L, Drahos L, Visnovitz T, Koltai E, Radák Z (2024) Effect of the 35 nm and 70 nm size exclusion chromatography (SEC) column and plasma storage time on separated extracellular vesicles. Curr Issues Mol Biol 46(5):4337–4357. 10.3390/cimb46050264 38785532 10.3390/cimb46050264 PMC11120626

[CR22] Hannum G, Guinney J, Zhao L, Zhang L, Hughes G, Sadda S, Klotzle B, Bibikova M, Fan JB, Gao Y, Deconde R, Chen M, Rajapakse I, Friend S, Ideker T, Zhang K (2013) Genome-wide methylation profiles reveal quantitative views of human aging rates. Mol Cell 49(2):359–367. 10.1016/j.molcel.2012.10.016 23177740 10.1016/j.molcel.2012.10.016 PMC3780611

[CR23] Horvath S (2013) DNA methylation age of human tissues and cell types. Genome Biol 14(10):R115. 10.1186/gb-2013-14-10-r115 24138928 10.1186/gb-2013-14-10-r115 PMC4015143

[CR24] Horvath S, Raj K (2018) DNA methylation-based biomarkers and the epigenetic clock theory of ageing. Nat Rev Genet 19(6):371–384. 10.1038/s41576-018-0004-3 29643443 10.1038/s41576-018-0004-3

[CR25] Huang KY, Upadhyay G, Ahn Y, Sakakura M, Pagan-Diaz GJ, Cho Y, Weiss AC, Huang C, Mitchell JW, Li J, Tan Y, Deng YH, Ellis-Mohr A, Dou Z, Zhang X, Kang S, Chen Q, Sweedler JV, Im SG, Bashir R, Chung HJ, Popescu G, Gillette MU, Gazzola M, Kong H (2024) Neuronal innervation regulates the secretion of neurotrophic myokines and exosomes from skeletal muscle. Proc National Acad Sci United States of Am 121(19):e2313590121. 10.1073/pnas.2313590121 10.1073/pnas.2313590121 PMC11087749 38683978

[CR26] Jokai M, Torma F, McGreevy KM, Koltai E, Bori Z, Babszki G, Bakonyi P, Gombos Z, Gyorgy B, Aczel D, Toth L, Osvath P, Fridvalszky M, Teglas T, Posa A, Kujach S, Olek R, Kawamura T, Seki Y, Suzuki K, Tanisawa K, Goto S, Kerepesi C, Boldogh I, Ba X, Davies KJA, Horvath S, Radak Z (2023) DNA methylation clock DNAmFitAge shows regular exercise is associated with slower aging and systemic adaptation. GeroScience 45(5):2805–2817. 10.1007/s11357-023-00826-1 37209203 10.1007/s11357-023-00826-1 PMC10643800

[CR27] Kaminsky LA, Arena R, Myers J (2015) Reference standards for cardiorespiratory fitness measured with cardiopulmonary exercise testing. Mayo Clin Proc 90(11):1515–1523. 10.1016/j.mayocp.2015.07.026 26455884 10.1016/j.mayocp.2015.07.026 PMC4919021

[CR28] Koltai E, Szabo Z, Atalay M, Boldogh I, Naito H, Goto S, Nyakas C, Radak Z (2009) Exercise alters SIRT1, SIRT6, NAD and NAMPT levels in skeletal muscle of aged rats. Mech Ageing Dev 131(1):21–28. 10.1016/j.mad.2009.11.002 19913571 10.1016/j.mad.2009.11.002 PMC2872991

[CR29] Koltai E, Hart N, Taylor AW, Goto S, Ngo JK, Davies KJ, Radak Z (2012) Age-associated declines in mitochondrial biogenesis and protein quality control factors are minimized by exercise training. Am J Physiol Regul Integr Comp Physiol 303(2):R127–R134. 10.1152/ajpregu.00337.2011 22573103 10.1152/ajpregu.00337.2011 PMC3404634

[CR30] Koncz A, Turiák L, Németh K, Lenzinger D, Bárkai T, Lőrincz P, Zelenyánszki H, Vukman KV, Buzás EI, Visnovitz T (2023) Endoplasmin is a hypoxia-inducible endoplasmic reticulum-derived cargo of extracellular vesicles released by cardiac cell lines. Membranes 13(4):431. 10.3390/membranes13040431 37103858 10.3390/membranes13040431 PMC10142439

[CR31] Lacroix R, Judicone C, Poncelet P, Robert S, Arnaud L, Sampol J, Dignat-George F (2012) Impact of pre-analytical parameters on the measurement of circulating microparticles: towards standardization of protocol. J Thromb Haemost 10(3):437–446. 10.1111/j.1538-7836.2011.04610.x 22212198 10.1111/j.1538-7836.2011.04610.x

[CR32] Lang JJ, Prince SA, Merucci K, Cadenas-Sanchez C, Chaput JP, Fraser BJ, Manyanga T, McGrath R, Ortega FB, Singh B, Tomkinson GR (2024) Cardiorespiratory fitness is a strong and consistent predictor of morbidity and mortality among adults: an overview of meta-analyses representing over 20.9 million observations from 199 unique cohort studies. Br J Sports Med 58(10):556–566. 10.1136/bjsports-2023-107849 38599681 10.1136/bjsports-2023-107849 PMC11103301

[CR33] Langfelder P, Horvath S (2008) WGCNA: an R package for weighted correlation network analysis. BMC Bioinformatics 9:559. 10.1186/1471-2105-9-559 19114008 10.1186/1471-2105-9-559 PMC2631488

[CR35] Liu YG, Jiang ST, Zhang JW, Zheng H, Zhang L, Zhao HT, Sang XT, Xu YY, Lu X (2024) Role of extracellular vesicle-associated proteins in the progression, diagnosis, and treatment of hepatocellular carcinoma. Cell Biosci 14(1):113. 10.1186/s13578-024-01294-6 39227992 10.1186/s13578-024-01294-6 PMC11373138

[CR36] Llorente A, Brokāne A, Mlynska A, Puurand M, Sagini K, Folkmane S, Hjorth M, Martin-Gracia B, Romero S, Skorinkina D, Čampa M, Cešeiko R, Romanchikova N, Kļaviņa A, Käämbre T, Linē A (2024) From sweat to hope: The role of exercise-induced extracellular vesicles in cancer prevention and treatment. J Extracell Vesicles 13(8):e12500. 10.1002/jev2.12500 39183543 10.1002/jev2.12500 PMC11345496

[CR37] Lou J, Wu J, Feng M, Dang X, Wu G, Yang H, Wang Y, Li J, Zhao Y, Shi C, Liu J, Zhao L, Zhang X, Gao F (2022) Exercise promotes angiogenesis by enhancing endothelial cell fatty acid utilization via liver-derived extracellular vesicle miR-122-5p. J Sport Health Sci 11(4):495–508. 10.1016/j.jshs.2021.09.009 34606978 10.1016/j.jshs.2021.09.009 PMC9338338

[CR38] Lu AT, Quach A, Wilson JG, Reiner AP, Aviv A, Raj K, Hou L, Baccarelli AA, Li Y, Stewart JD, Whitsel EA, Assimes TL, Ferrucci L, Horvath S (2019) DNA methylation GrimAge strongly predicts lifespan and healthspan. Aging 11(2):303–327. 10.18632/aging.101684 30669119 10.18632/aging.101684 PMC6366976

[CR39] Lu AT, Binder AM, Zhang J, Yan Q, Reiner AP, Cox SR, Corley J, Harris SE, Kuo PL, Moore AZ, Bandinelli S, Stewart JD, Wang C, Hamlat EJ, Epel ES, Schwartz JD, Whitsel EA, Correa A, Ferrucci L, Marioni RE, Horvath S (2022) DNA methylation GrimAge version 2. Aging 14(23):9484–9549. 10.18632/aging.204434 36516495 10.18632/aging.204434 PMC9792204

[CR40] Mazzocco M, Arrigo P, Egeo A, Maffei M, Vergano A, Di Lisi R, Ghiotto F, Ciccone E, Cinti R, Ravazzolo R, Scartezzini P (2001) A novel human homologue of the SH3BGR gene encodes a small protein similar to Glutaredoxin 1 of Escherichia coli. Biochem Biophys Res Commun 285(2):540–545. 10.1006/bbrc.2001.5169 11444877 10.1006/bbrc.2001.5169

[CR41] McGreevy KM, Radak Z, Torma F, Jokai M, Lu AT, Belsky DW, Binder A, Marioni RE, Ferrucci L, Pośpiech E, Branicki W, Ossowski A, Sitek A, Spólnicka M, Raffield LM, Reiner AP, Cox S, Kobor M, Corcoran DL, Horvath S (2023) DNAmFitAge: biological age indicator incorporating physical fitness. Aging 15(10):3904–3938. 10.18632/aging.204538 36812475 10.18632/aging.204538 PMC10258016

[CR42] McIlvenna LC, Whitham M (2023) Exercise, healthy ageing, and the potential role of small extracellular vesicles. J Physiol 601(22):4937–4951. 10.1113/JP282468 35388915 10.1113/JP282468 PMC10952297

[CR43] Mi H, Muruganujan A, Casagrande JT, Thomas PD (2013) Large-scale gene function analysis with the PANTHER classification system. Nat Protoc 8(8):1551–1566. 10.1038/nprot.2013.092 23868073 10.1038/nprot.2013.092 PMC6519453

[CR44] Nederveen JP, Warnier G, di Carlo A, Nilsson MI, Tarnopolsky MA (2021) Extracellular vesicles and exosomes: insights from exercise science. Front Physiol. 10.3389/fphys.2020.604274 33597890 10.3389/fphys.2020.604274 PMC7882633

[CR45] Pepe J, Rossi M, Battafarano G, Vernocchi P, Conte F, Marzano V, Mariani E, Mortera SL, Cipriani C, Rana I, Buonuomo PS, Bartuli A, de Martino V, Pelle S, Pascucci L, Toniolo RM, Putignani L, Minisola S, del Fattore A (2022) Characterization of extracellular vesicles in osteoporotic patients compared to osteopenic and healthy controls. J Bone and Miner Res : Official J Am Soc for Bone and Miner Res 37(11):2186–2200. 10.1002/jbmr.4688 10.1002/jbmr.4688 PMC10086946 36053959

[CR46] Radak Z, Chung HY, Goto S (2008) Systemic adaptation to oxidative challenge induced by regular exercise. Free Radical Biol Med 44(2):153–159. 10.1016/j.freeradbiomed.2007.01.029 18191751 10.1016/j.freeradbiomed.2007.01.029

[CR47] Radak Z, Pan L, Zhou L, Mozaffaritabar S, Yaodong G, Pinho RA, Zheng X, Ba X, Boldogh I (2024) Epigenetic and “redoxogenetic” adaptation to physical exercise. Free Radical Biol Med 210(65):74. 10.1016/j.freeradbiomed.2023.11.005 10.1016/j.freeradbiomed.2023.11.005 37977212

[CR48] Radák Z, Aczél D, Fejes I, Mozaffaritabar S, Pavlik G, Komka Z, Balogh L, Babszki Z, Babszki G, Koltai E, McGreevy KM, Gordevicius J, Horvath S (2024) Kerepesi C (2024b) slowed epigenetic aging in olympic champions compared to non-champions. Geroscience. 10.1007/s11357-024-01440-5 39601999 10.1007/s11357-024-01440-5 PMC11978583

[CR49] Romani R, Talesa VN, Antognelli C (2022) The glyoxalase system is a novel cargo of amniotic fluid stem-cell-derived extracellular vesicles. Antioxidants (Basel, Switzerland). 10.3390/antiox11081524 36009243 10.3390/antiox11081524 PMC9405222

[CR50] Ross R, Blair SN, Arena R, Church TS, Després J-P, Franklin BA, Haskell WL, Kaminsky LA, Levine BD, Lavie CJ, Myers J, Niebauer J, Sallis R, Sawada SS, Sui X, Wisløff U, American Heart Association Physical Activity Committee of the Council on Lifestyle and Cardiometabolic Health; Council on Clinical Cardiology; Council on Epidemiology and Prevention; Council on Cardiovascular and Stroke Nursing; Council on Functional Genomics and Translational Biology; Stroke Council (2016) Importance of assessing cardiorespiratory fitness in clinical practice: a case for fitness as a clinical vital sign: a scientific statement from the american heart association. Circulation 134(24):e653–e699. 10.1161/CIR.0000000000000461 27881567 10.1161/CIR.0000000000000461

[CR51] Sánchez-Sánchez JL, Lu WH, Gallardo-Gómez D, Del Pozo CB, de Souto BP, Lucia A, Valenzuela PL (2024) Association of intrinsic capacity with functional decline and mortality in older adults: a systematic review and meta-analysis of longitudinal studies. Lancet Healthy Longev 5(7):e480–e492. 10.1016/S2666-7568(24)00092-8 38945130 10.1016/S2666-7568(24)00092-8

[CR52] Sasaki K, Takano S, Tomizawa S, Miyahara Y, Furukawa K, Takayashiki T, Kuboki S, Takada M, Ohtsuka M (2021) C4b-binding protein α-chain enhances antitumor immunity by facilitating the accumulation of tumor-infiltrating lymphocytes in the tumor microenvironment in pancreatic cancer. J Exp Clin Cancer Res 40(1):212. 10.1186/s13046-021-02019-0 34167573 10.1186/s13046-021-02019-0 PMC8228942

[CR53] Seki Y, Aczel D, Torma F, Jokai M, Boros A, Suzuki K, Higuchi M, Tanisawa K, Boldogh I, Horvath S, Radak Z (2023) No strong association among epigenetic modifications by DNA methylation, telomere length, and physical fitness in biological aging. Biogerontology 24(2):245–255. 10.1007/s10522-022-10011-0 36592269 10.1007/s10522-022-10011-0 PMC10006047

[CR54] Sheta M, Taha EA, Lu Y, Eguchi T (2023) Extracellular vesicles: new classification and tumor immunosuppression. Biology 12(1):110. 10.3390/biology12010110 36671802 10.3390/biology12010110 PMC9856004

[CR55] Simpson RJ, Lowder TW, Spielmann G, Bigley AB, LaVoy EC, Kunz H (2012) Exercise and the aging immune system. Ageing Res Rev 11(3):404–420. 10.1016/j.arr.2012.03.003 22465452 10.1016/j.arr.2012.03.003

[CR56] Singh S, Kumar P, Padwad YS, Jaffer FA, Reed GL (2024) Targeting fibrinolytic inhibition for venous thromboembolism treatment: overview of an emerging therapeutic approach. Circulation 150(11):884–898. 10.1161/CIRCULATIONAHA.124.069728 39250537 10.1161/CIRCULATIONAHA.124.069728 PMC11433585

[CR57] Suades R, Padró T, Vilahur G, Badimon L (2022) Platelet-released extracellular vesicles: the effects of thrombin activation. Cell and Mol Life Sci: CMLS 79(3):190. 10.1007/s00018-022-04222-4 35288766 10.1007/s00018-022-04222-4 PMC8920058

[CR58] Théry C, Amigorena S, Raposo G, Clayton A (2006) Isolation and characterization of exosomes from cell culture supernatants and biological fluids. Curr Protoc Cell Biol. 10.1002/0471143030.cb0322s30 18228490 10.1002/0471143030.cb0322s30

[CR59] Théry C, Witwer KW, Aikawa E, Alcaraz MJ, Anderson JD, Andriantsitohaina R, Antoniou A, Arab T, Archer F, Atkin-Smith GK, Ayre DC, Bach J, Bachurski D, Baharvand H, Balaj L, Baldacchino S, Bauer NN, Baxter AA, Bebawy M et al (2018) Minimal information for studies of extracellular vesicles 2018 (MISEV2018): a position statement of the international society for extracellular vesicles and update of the MISEV2014 guidelines. J Extracell Vesicles. 10.1080/20013078.2018.1535750 30637094 10.1080/20013078.2018.1535750 PMC6322352

[CR60] Turiák L, Misják P, Szabó TG, Aradi B, Pálóczi K, Ozohanics O, Drahos L, Kittel A, Falus A, Buzas EI, Vékey K (2011) Proteomic characterization of thymocyte-derived microvesicles and apoptotic bodies in BALB/c mice. J Proteomics 74(10):2025–2033. 10.1016/j.jprot.2011.05.023 21635979 10.1016/j.jprot.2011.05.023

[CR61] Tyanova S, Temu T, Cox J (2016) The MaxQuant computational platform for mass spectrometry-based shotgun proteomics. Nat Protoc 11(12):2301–2319. 10.1038/nprot.2016.136 27809316 10.1038/nprot.2016.136

[CR62] Upadhya D, Shetty AK (2024) MISEV2023 provides an updated and key reference for researchers studying the basic biology and applications of extracellular vesicles. Stem Cells Transl Med 13(9):848–850. 10.1093/stcltm/szae052 39028333 10.1093/stcltm/szae052 PMC11386207

[CR63] van Niel G, D’Angelo G, Raposo G (2018) Shedding light on the cell biology of extracellular vesicles. Nat Rev Mol Cell Biol 19(4):213–228. 10.1038/nrm.2017.125 29339798 10.1038/nrm.2017.125

[CR64] Wang Y, Liu Y, Zhang S, Li N, Xing C, Wang C, Wang J, Wei M, Yang G, Yuan L (2023) Exercise improves metabolism and alleviates atherosclerosis via muscle-derived extracellular vesicles. Aging and Dis 14(3):952. 10.14336/AD.2022.1131 10.14336/AD.2022.1131 PMC10187707 37191422

[CR65] Welsh JA, Goberdhan DCI, O’Driscoll L, Buzas EI, Blenkiron C, Bussolati B, Cai H, Di Vizio D, Driedonks TAP, Erdbrügger U, Falcon-Perez JM, Qing-Ling F, Hill AF, Lenassi M, Lim SK, Mahoney MG, Mohanty S, Möller A, Nieuwland R, Ochiya T, Sahoo S, Torrecilhas AC, Zheng L, Zijlstra A, Abuelreich S, Bagabas R, Bergese P, Bridges EM, Brucale M, Burger D, Carney RP, Cocucci E, Crescitelli R, Hanser E, Harris AL, Haughey NJ, Hendrix A, Ivanov AR, Jovanovic-Talisman T, Kruh-Garcia NA, Faustino VK-L, Kyburz D, Lässer C, Lennon KM, Lötvall J, Maddox AL, Martens-Uzunova ES, Mizenko RR, Newman LA, Ridolfi A, Rohde E, Rojalin T, Rowland A, Saftics A, Sandau US, Saugstad JA, Shekari F, Swift S, Ter-Ovanesyan D, Tosar JP, Useckaite Z, Valle F, Varga Z, van der Pol E, van Herwijnen MJC, Wauben MHM, Wehman AM, Williams S, Zendrini A, Zimmerman AJ, Théry C, Witwer KW (2024) Minimal information for studies of extracellular vesicles (MISEV2023): From basic to advanced approaches. J Extracell Vesicles. 10.1002/jev2.12404 39140467 10.1002/jev2.12498 PMC11322860

[CR66] Zhang B, Zhao J, Jiang M, Peng D, Dou X, Song Y, Shi J (2022) The potential role of gut microbial-derived exosomes in metabolic-associated fatty liver disease: implications for treatment. Front Immunol 13:893617. 10.3389/fimmu.2022.893617 35634340 10.3389/fimmu.2022.893617 PMC9131825

