# Supplementary material for: The protein cargo of extracellular vesicles correlates with the epigenetic aging clock of exercise sensitive DNAmFitAge
Source: Biogerontology. 2025 Jan 8;26(1):35. doi: 10.1007/s10522-024-10177-9 (PMC11711255; doi:10.1007/s10522-024-10177-9)
Supplement: Supplementary file 1 — Supplementary file1 (PDF 208 KB) [file 10522_2024_10177_MOESM1_ESM.pdf]

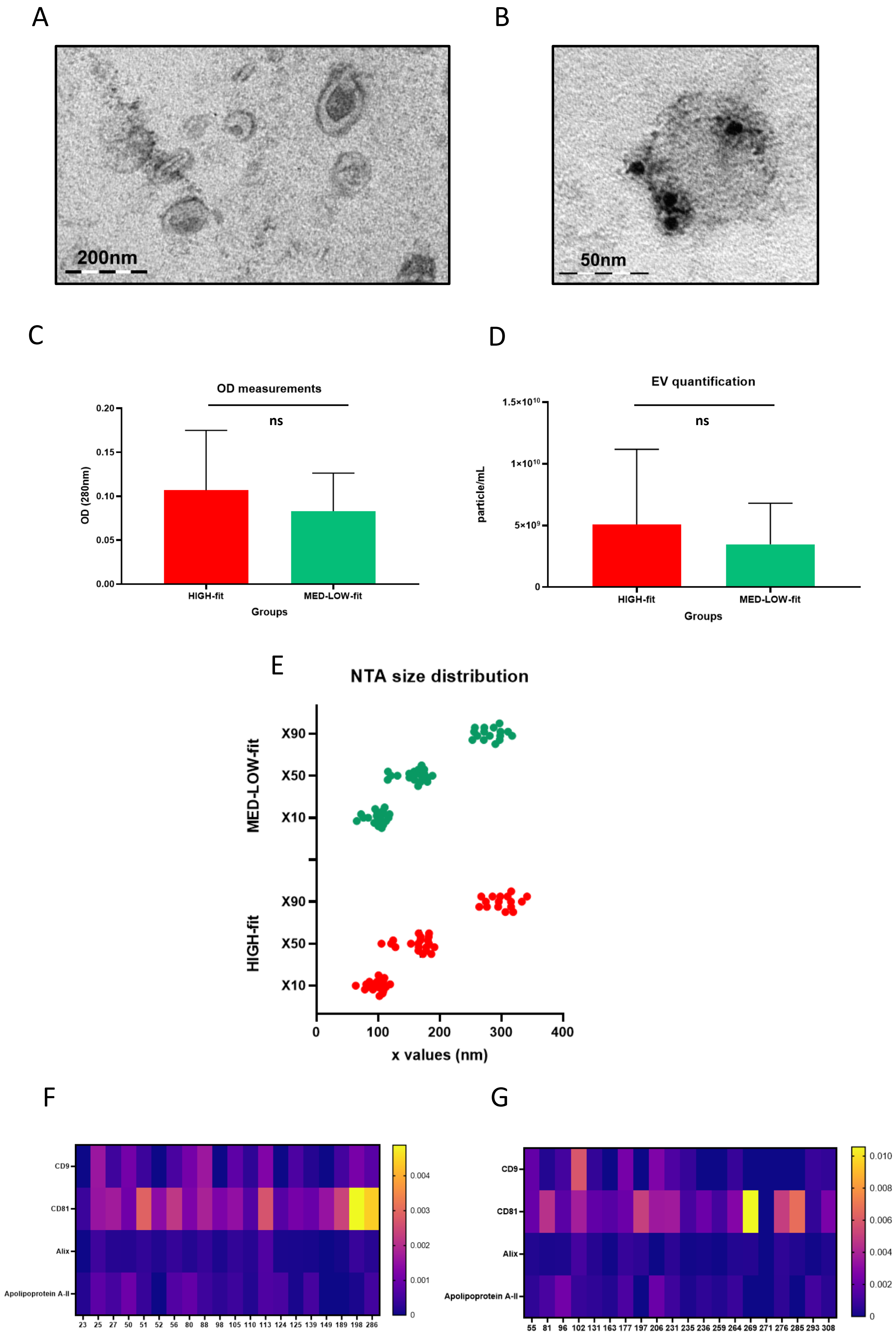

Supplementary Figure S1. Analysis of sEVs isolated from High-fit (red) and Med-Low-fit (green) groups. (**A**): TEM images showing the morphology of sEVs after SEC+UC. (**B**): Immuno TEM images of sEVs after SEC+UC. Scale bar sizes are 200 and 50 nm for (A) and (B). Images indicate the presence of vesicles. Average results of sEV characterisation isolated from High-fit (n=20) and Med-Low-fit (n=20). (**C**): The X axis represents the two group, the Y-axis shows Nanodrop absorbance results at OD280. (**D**): The X axis represents the two group, the Y-axis shows NTA results in particle/mL. (**E**): The NTA size distribution (X10, X50, X90) of HIGH-fit (n=20) and Med-Low-fit (n=20) groups. The heatmap shows the relative intensity of vesicle markers in (**F**): High-Fit and (**G**): Medium-Low-fit samples compared to total protein intensity, as determined by mass spectrometry data. The x-axis represents the sample numbers, while the y-axis indicates the vesicle markers.
