# Supplementary material for: The protein cargo of extracellular vesicles correlates with the epigenetic aging clock of exercise sensitive DNAmFitAge
Source: Biogerontology. 2025 Jan 8;26(1):35. doi: 10.1007/s10522-024-10177-9 (PMC11711255; doi:10.1007/s10522-024-10177-9)
Supplement: Supplementary file 2 — Supplementary file2 (PDF 421 KB) [file 10522_2024_10177_MOESM2_ESM.pdf]

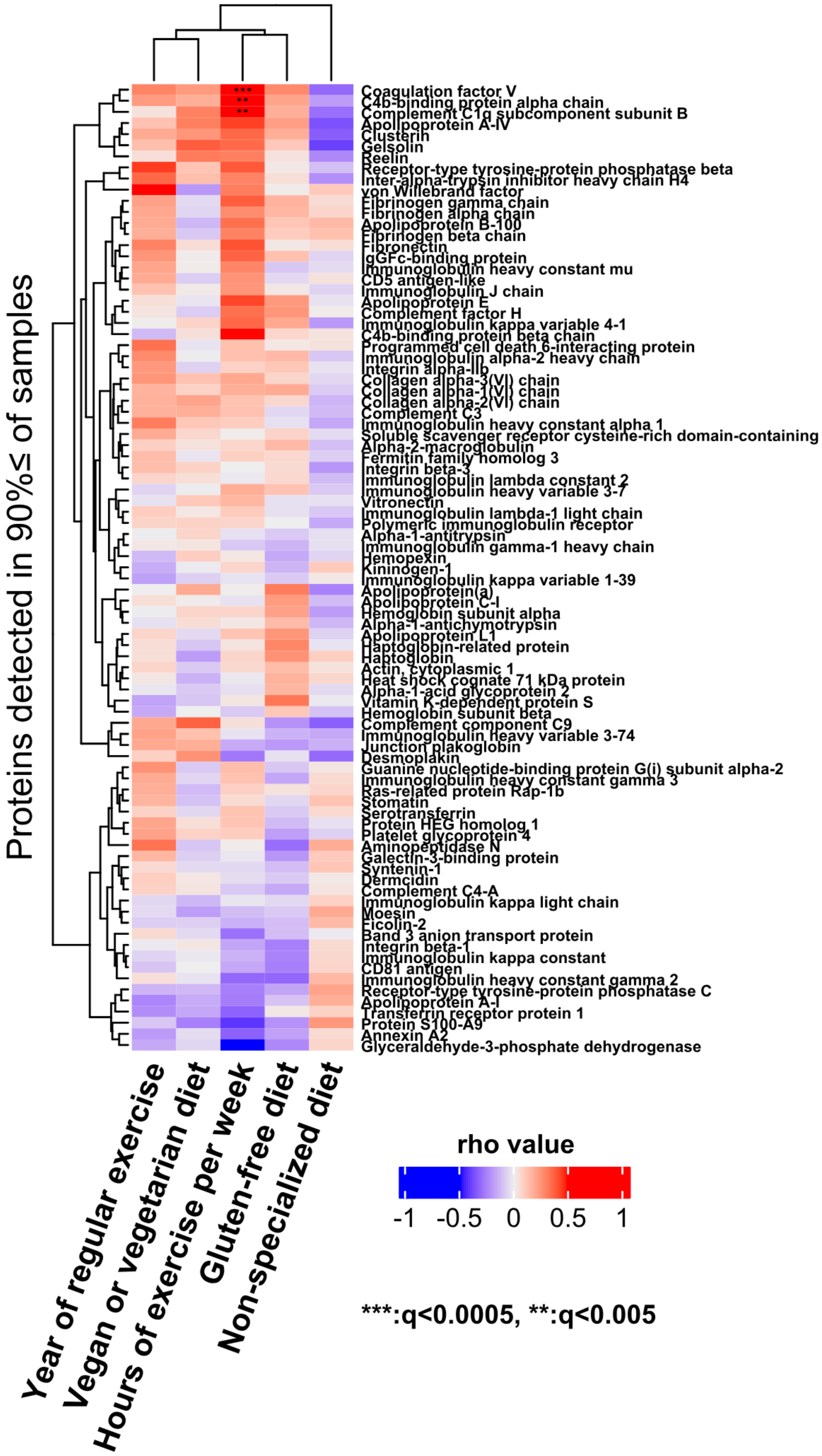

Supplementary Figure S2. Heat map depicting the correlation between protein mass spectrometry normalized intensity variables and lifestyle markers. These markers include Year of regular exercise, Vegan or vegetarian diet, Hours of exercise per week, Gluten-free diet, Non-specialized diet.
