# Supplementary material for: The protein cargo of extracellular vesicles correlates with the epigenetic aging clock of exercise sensitive DNAmFitAge
Source: Biogerontology. 2025 Jan 8;26(1):35. doi: 10.1007/s10522-024-10177-9 (PMC11711255; doi:10.1007/s10522-024-10177-9)
Supplement: Supplementary file 3 — Supplementary file3 (DOCX 21 KB) [file 10522_2024_10177_MOESM3_ESM.docx]

**Supplementary Tables**

**Supplementary Table S1.** Antibodies for TEM, which used in the study.

| **Antibody’s name** | **Manufacturer** | **Cat. number** |
| --- | --- | --- |
| Anti–CD9 | Abcam | ab92726 |
| Goat anti–rabbit IgG 5 nm gold | Sigma | G7277 |

**Supplementary Table S2.** Characteristics of subjects and mean values of measured parameters.

|  |  | **High-fit**  **(n=20)** | **Med-Low-fit**  **(n=20)** |
| --- | --- | --- | --- |
| Age | Mean | 57.650 | 57.500 |
|  | SD | 9.783 | 9.583 |
| VO_2_max (mL/min/kg) | Mean | 44.721 | 33.906 |
|  | SD | 6.564 | 8.744 |
| Body mass (kg) | Mean | 68.255 | 66.990 |
|  | SD | 9.054 | 8.618 |
| Body high (cm) | Mean | 169.395 | 164.305 |
|  | SD | 4.081 | 6.443 |
| BMI | Mean | 23.460 | 24.890 |
|  | SD | 3.129 | 3.282 |
| JumpMax (cm) | Mean | 29.455 | 24.620 |
|  | SD | 5.686 | 6.567 |
| GripMax (kg/BM) | Mean | 37.010 | 28.375 |
|  | SD | 4.402 | 5.254 |
| LDL | Mean | 3.057 | 3.304 |
|  | SD | 0.497 | 0.550 |
| HDL | Mean | 1.971 | 1.672 |
|  | SD | 0.378 | 0.366 |

|  |  |  |  |
| --- | --- | --- | --- |
|  |  | **High-fit**  **(n=20)** | **Med-Low-fit**  **(n=20)** |
| Cognitive | Mean | 6.600 | 6.150 |
|  | SD | 1.273 | 0.988 |
| AgeAccelPheno | Mean | 0.340 | -1.521 |
|  | SD | 5.312 | 6.340 |
| AgeAccelGrim | Mean | -1.444 | -0.866 |
|  | SD | 3.092 | 3.516 |
| AgeAccelFit | Mean | -0.586 | 0.582 |
|  | SD | 3.350 | 3.170 |
