# Supplementary material for: The protein cargo of extracellular vesicles correlates with the epigenetic aging clock of exercise sensitive DNAmFitAge
Source: Biogerontology. 2025 Jan 8;26(1):35. doi: 10.1007/s10522-024-10177-9 (PMC11711255; doi:10.1007/s10522-024-10177-9)
Supplement: Supplementary file 4 — Supplementary file4 (DOCX 16 KB) [file 10522_2024_10177_MOESM4_ESM.docx]

S1: Pre-Analytical assessment of PFP

Of the 40 prepared PFP samples, 500 μL were allocated for pre-analytical assessment. Platelet counts (million cells/L; mean ± SD = 0.73 ± 0.85) and haemoglobin concentrations (g/L; mean ± SD = 0.30 ± 0.46) were initially quantified using a haematology analyser (Sysmex XE2100). After 0.8 μm filtration during sEV isolation, platelet counts in the PFP were reassessed with the same analyser, showing “zero million cells/L” in all samples (n = 40). Subsequently, an ELISA plate reader was utilised to measure absorbance at five wavelengths (450 nm, 492 nm, 540 nm, 620 nm, and 690 nm). Haemolysis was detected at 540 nm (mean ± SD = 0.091 ± 0.017). Icterus was measured at 450 nm (mean ± SD = 0.335 ± 0.059) and 492 nm (mean ± SD = 0.259 ± 0.048), while lipemia was assessed at 620 nm (mean ± SD = 0.063 ± 0.012) and 690 nm (mean ± SD = 0.055 ± 0.016).

S2: Analysis of sEVs

Based on the pre-analytical results, sEVs were isolated from all 40 samples using 70 nm SEC columns. As NTA method is unable to distinguish between "vesicular" and "non-vesicular" particles, TEM is also needed to identify vesicles. Morphological characterisation and immuno-TEM imaging were conducted using an anti-CD9 antibody (Abcam, Cambridge, UK) to label the EVs within the samples (Supplementary Figure S1A-B). Analysis by TEM showed that the samples contained EVs with a typical cup-shaped structure.

We used NTA to examine the size distribution and concentration of sEVs (Supplementary Figure 1D-E). Based on size differences, we observed three slightly overlapping populations: “plasma lipoprotein particles” ("PLPs"), sEVs and medium-sized EVs (mEVs). The exclusion criteria for concentration was 1 x 10^9^ particle/mL. The mean particle concentration was 5.08 × 10^9 particles/mL in the High-fit group and 3.46 × 10^9 particles/mL in the Med-Low-fit group. For the High-fit group, the NTA mean sizes (nm) were as follows: X10 values ± SD = 98.0 ± 13.6; X50 values ± SD = 163.2 ± 24.4; and X90 values ± SD = 277.9 ± 51.7. For the Med-Low-fit group, the NTA mean sizes (nm) were: X10 values ± SD = 98.7 ± 14.4; X50 values ± SD = 158.1 ± 21.6 and X90 values ± SD = 265.4 ± 39.6.

The protein absorbance was measured at 280 nm using a NanoDrop instrument. The mean protein absorbance ± SD was 0.107 ± 0.068 in the High-fit group and 0.083 ± 0.043 in the Med-Low-fit group. In all cases, the total protein concentration was below the exclusion criterion of 0.75 mg/mL. The data are presented in Supplementary Figure S1C. In all instances, no significant differences were detected between the groups.

MS is widely used in EV studies to detect and characterise EV-associated proteins in different samples. The heatmap figure (Supplementary Figure S1F-G) shows the intensity of vesicle markers (CD9, CD81, Alix) in samples from the High-fit and Med-Low-fit groups, relative to the total protein intensity based on MS data. The presence of CD81 was detected in 39 out of 40 samples. We also detected CD9 (27 out of 40 samples) and Alix (37 out of 40 samples) vesicle markers. Apolipoproteins can co-isolate with vesicles in human plasma samples. This is indicated by the detection of Apolipoprotein A-II (ApoA-II) protein in the samples. The analysis of protein markers was conducted in accordance with the MISEV2023 guidelines.

The GO-term enrichment results (Figure 1B, Figure 4A-B) further validate the vesicle content of the samples by highlighting the enrichment of gene ontology terms associated with vesicle-related biological processes, molecular functions, and cellular components.

S3: HPLC and MS acquisition parameters

The analytes were pre-concentrated on a PepMap RSLC C18 (0.3 x 5 mm) column using 0.1% FA/1% ACN at a flow rate of 30 µL/min for 3 minutes and separated on a Thermo Scientific EASY-spray PepMap RSLC C18 (75 µm x 50 cm) analytical column using a linear gradient elution. Eluent A was 0.1% FA/H_2_O and eluent B was 0.1% FA/ACN. Flow rate was 250 nL/min, column temperature was set to 40 °C. The initial 3% B was linearly increased to 40% in 120 min. Ionization was carried out using ESI (electrospray ionization) at 2 kV in positive ionization mode. DDA (data-dependent analysis) was used with a full MS precursor scan from 350 to 2000 m/z at a resolution of 60000. Dependent MS/MS scans of the top 20 most intense precursor ions were done using 30% HCD collision energy, isolation window of 1 m/z and a resolution of 15000. Before MS/MS scans, precursor ions were filtered based on intensity (1×10^3^), charge state (2-4) and a dynamic exclusion was also employed with a duration of 60s.
